# (Phen­yl)(1-phenyl­sulfonyl-1*H*-indol-3-yl)methanone

**DOI:** 10.1107/S1600536810042261

**Published:** 2010-10-23

**Authors:** G. Chakkaravarthi, R. Panchatcharam, V. Dhayalan, A. K. Mohanakrishnan, V. Manivannan

**Affiliations:** a Department of Physics, CPCL Polytechnic College, Chennai 600 068, India; b Department of Research and Development, PRIST University, Vallam, Thanjavur 613 403, Tamil Nadu, India; c Department of Organic Chemistry, University of Madras, Guindy Campus, Chennai 600 025, India

## Abstract

In the title compound, C_21_H_15_NO_3_S, the sulfonyl-bound phenyl ring forms a dihedral angle of 86.28 (5)° with the indole ring system. The mol­ecular structure is stabilized by intra­molecular C—H⋯O hydrogen bonds. The crystal packing is stabilized by weak inter­molecular C—H⋯O and C—H⋯π inter­actions.

## Related literature

For the structures of closely related compounds, see: Chakkaravarthi *et al.* (2007 ▶, 2008 ▶).
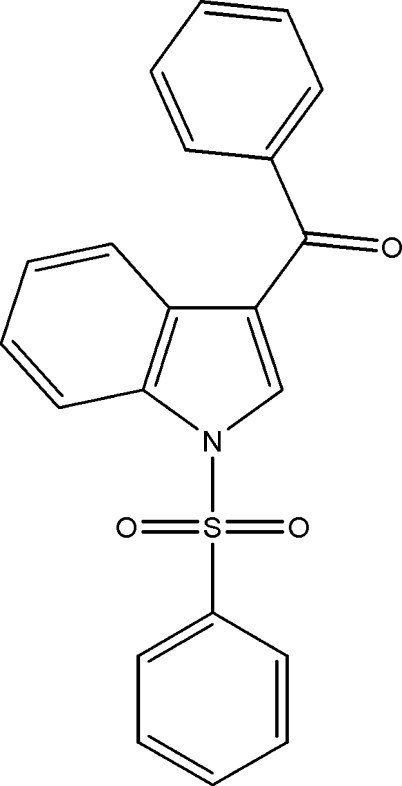


## Experimental

### 

#### Crystal data

C_21_H_15_NO_3_S
*M* *_r_* = 361.40
Triclinic, 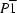

*a* = 7.567 (1) Å
*b* = 10.571 (2) Å
*c* = 12.083 (3) Å
α = 66.302 (2)°
β = 80.740 (1)°
γ = 78.403 (1)°
*V* = 863.5 (3) Å^3^
*Z* = 2
Mo *K*α radiation
μ = 0.21 mm^−1^
*T* = 295 K
0.24 × 0.22 × 0.20 mm

#### Data collection

Bruker Kappa APEXII diffractometer
Absorption correction: multi-scan (*SADABS*; Sheldrick, 1996 ▶) *T*_min_ = 0.952, *T*_max_ = 0.960
15638 measured reflections
4276 independent reflections
3187 reflections with *I* > 2σ(*I*)
*R*_int_ = 0.026
Standard reflections: 0

#### Refinement

*R*[*F* ^2^ > 2σ(*F* ^2^)] = 0.037
*wR*(*F* ^2^) = 0.109
*S* = 1.01
4276 reflections
235 parameters
2 restraints
H-atom parameters constrained
Δρ_max_ = 0.21 e Å^−3^
Δρ_min_ = −0.29 e Å^−3^

### 

Data collection: *APEX2* (Bruker, 2004 ▶); cell refinement: *SAINT* (Bruker, 2004 ▶); data reduction: *SAINT*; program(s) used to solve structure: *SHELXS97* (Sheldrick, 2008 ▶); program(s) used to refine structure: *SHELXL97* (Sheldrick, 2008 ▶); molecular graphics: *PLATON* (Spek, 2009 ▶); software used to prepare material for publication: *SHELXL97*.

## Supplementary Material

Crystal structure: contains datablocks I, global. DOI: 10.1107/S1600536810042261/bt5379sup1.cif

Structure factors: contains datablocks I. DOI: 10.1107/S1600536810042261/bt5379Isup2.hkl

Additional supplementary materials:  crystallographic information; 3D view; checkCIF report

## Figures and Tables

**Table 1:** Hydrogen-bond geometry (Å, °) *Cg*1 is the centroid of the C16–C21 ring.

*D*—H⋯*A*	*D*—H	H⋯*A*	*D*⋯*A*	*D*—H⋯*A*
C13—H13⋯O1	0.93	2.42	2.999 (2)	120
C6—H6⋯O2^i^	0.93	2.58	3.493 (2)	167
C7—H7⋯O2^i^	0.93	2.54	3.429 (2)	160
C4—H4⋯*Cg*1^ii^	0.93	2.98	3.774 (3)	144

Symmetry codes: (i) 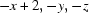; (ii) 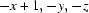.

**Fig. 1.**
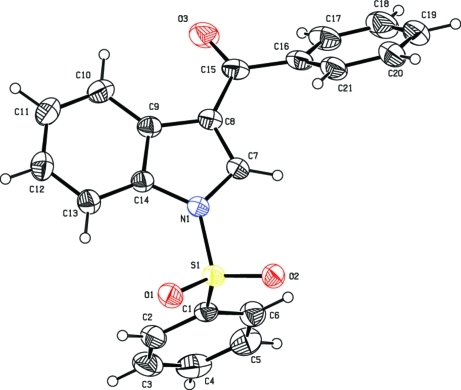
The molecular structure of (I), with atom labels and 30% probability displacement ellipsoids.

**Fig. 2.**
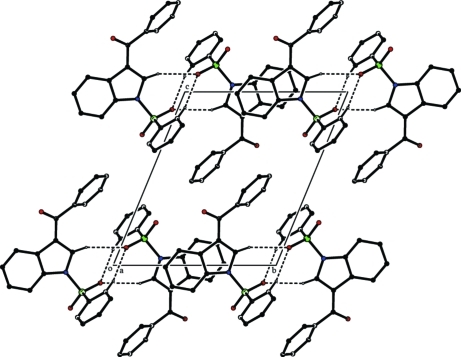
The packing of (I), viewed down the a axis. H-bonds are shown as dashed lines; H atoms not involved in hydrogen bonding have been omitted.

## supplementary crystallographic information

### Comment

The geometric parameters of the molecule of (I) (Fig. 1) agree well with the reported values of similar structures (Chakkaravarthi *et al.*, 2007; Chakkaravarthi *et al.*, 2008). The phenyl rings C1—C6 and C16—C21 form the dihedral angles of 86.28 (5)° and 51.91 (5)°, respectively with the indole ring system. The mean planes of the two phenyl rings are inclined at an angle of 42.16 (6)°.

The sum of the bond angles around N1 [358.53°] indicates that N1 atom is *sp*^2^ hybridized. The molecular structure is stabilized by intra molecular C—H···O hydrogen bonds and the crystal packing is stabilized by weak intermolecular C—H···O and C—H···π [C4—H4···*Cg*1 (1 - *x*, -*y*, -*z*) distance of 3.774 (3)Å (*Cg*1 is the centroid of the ring defined by the atoms C16—C21)] interactions.

### Experimental

To a solution of 1-phenylsulfonyl-(1*H*-indol-3-yl)(phenyl)methanol (1 g, 2.75 mmol) in dry 1,2-Dichloroethane (30 ml), manganese dioxide (6 g. 68.96 mmol) was added then stirred at room temperature for 4 h and then refluxed for 3 h. Then the resulting solution was passed through celite pad and washed with DCM (2 *x* 30 ml). Removal of solvent followed by crystallization from methanol afforded the compound as a colourless crystal.

### Refinement

H atoms were positioned geometrically and refined using riding model with C—H = 0.93 Å and *U*_iso_(H) = 1.2Ueq(C) for aromatic C—H. The components of the anisotropic displacement parameters in direction of the bond of C3and C4; C18 and C19 were restrained to be equal within an effective standard deviation of 0.001 using the DELU command in *SHELXL* (Sheldrick, 2008).

### Crystal data

**Table:**

C_21_H_15_NO_3_S	*Z* = 2
*M**_r_* = 361.40	*F*(000) = 376
Triclinic, *P*1	*D*_x_ = 1.390 Mg m^−^^3^
Hall symbol: -P 1	Mo *K*α radiation, λ = 0.71073 Å
*a* = 7.567 (1) Å	Cell parameters from 6196 reflections
*b* = 10.571 (2) Å	θ = 2.2–27.1°
*c* = 12.083 (3) Å	µ = 0.21 mm^−^^1^
α = 66.302 (2)°	*T* = 295 K
β = 80.740 (1)°	Block, colourless
γ = 78.403 (1)°	0.24 × 0.22 × 0.20 mm
*V* = 863.5 (3) Å^3^	

### Data collection

**Table:**

Bruker Kappa APEXII diffractometer	4276 independent reflections
Radiation source: fine-focus sealed tube	3187 reflections with *I* > 2σ(*I*)
graphite	*R*_int_ = 0.026
ω and φ scans	θ_max_ = 28.3°, θ_min_ = 1.9°
Absorption correction: multi-scan (*SADABS*; Sheldrick, 1996)	*h* = −10→10
*T*_min_ = 0.952, *T*_max_ = 0.960	*k* = −14→13
15638 measured reflections	*l* = −16→16

### Refinement

**Table:**

Refinement on *F*^2^	Primary atom site location: structure-invariant direct methods
Least-squares matrix: full	Secondary atom site location: difference Fourier map
*R*[*F*^2^ > 2σ(*F*^2^)] = 0.037	Hydrogen site location: inferred from neighbouring sites
*wR*(*F*^2^) = 0.109	H-atom parameters constrained
*S* = 1.01	*w* = 1/[σ^2^(*F*_o_^2^) + (0.0543*P*)^2^ + 0.1447*P*] where *P* = (*F*_o_^2^ + 2*F*_c_^2^)/3
4276 reflections	(Δ/σ)_max_ < 0.001
235 parameters	Δρ_max_ = 0.21 e Å^−^^3^
2 restraints	Δρ_min_ = −0.29 e Å^−^^3^

### Fractional atomic coordinates and isotropic or equivalent isotropic displacement parameters (Å^2^)

**Table:**

	*x*	*y*	*z*	*U*_iso_*/*U*_eq_	
C1	0.7233 (2)	0.05722 (16)	0.20701 (13)	0.0494 (4)	
C2	0.6154 (3)	0.0869 (2)	0.29978 (15)	0.0648 (5)	
H2	0.6523	0.1388	0.3360	0.078*	
C3	0.4508 (3)	0.0376 (2)	0.33759 (18)	0.0791 (5)	
H3	0.3759	0.0567	0.3997	0.095*	
C4	0.3975 (3)	−0.0394 (2)	0.2838 (2)	0.0806 (6)	
H4	0.2866	−0.0718	0.3097	0.097*	
C5	0.5061 (3)	−0.0687 (2)	0.1928 (2)	0.0757 (5)	
H5	0.4690	−0.1215	0.1575	0.091*	
C6	0.6701 (2)	−0.02074 (19)	0.15289 (17)	0.0615 (4)	
H6	0.7440	−0.0403	0.0906	0.074*	
C14	0.80636 (19)	0.40437 (16)	0.04797 (13)	0.0448 (3)	
C13	0.7914 (2)	0.44779 (18)	0.14366 (15)	0.0547 (4)	
H13	0.8305	0.3873	0.2188	0.066*	
C12	0.7164 (2)	0.5838 (2)	0.12251 (18)	0.0641 (5)	
H12	0.7018	0.6157	0.1853	0.077*	
C11	0.6617 (3)	0.6751 (2)	0.00966 (19)	0.0681 (5)	
H11	0.6130	0.7671	−0.0019	0.082*	
C10	0.6778 (2)	0.63262 (18)	−0.08489 (17)	0.0609 (4)	
H10	0.6412	0.6948	−0.1603	0.073*	
C9	0.75050 (19)	0.49375 (16)	−0.06607 (14)	0.0472 (3)	
C8	0.7882 (2)	0.41551 (16)	−0.14359 (13)	0.0474 (3)	
C7	0.86292 (19)	0.28383 (16)	−0.07641 (12)	0.0469 (3)	
H7	0.8982	0.2113	−0.1040	0.056*	
C15	0.7413 (2)	0.46868 (18)	−0.26928 (14)	0.0546 (4)	
C16	0.8200 (2)	0.39033 (18)	−0.34881 (13)	0.0553 (4)	
C21	0.9990 (3)	0.32957 (19)	−0.35204 (14)	0.0634 (5)	
H21	1.0737	0.3291	−0.2978	0.076*	
C20	1.0679 (3)	0.2692 (2)	−0.43577 (17)	0.0807 (6)	
H20	1.1893	0.2301	−0.4391	0.097*	
C19	0.9549 (4)	0.2676 (2)	−0.51451 (18)	0.0891 (6)	
H19	1.0000	0.2259	−0.5700	0.107*	
C18	0.7771 (4)	0.3274 (3)	−0.51087 (19)	0.0900 (6)	
H18	0.7017	0.3266	−0.5642	0.108*	
C17	0.7103 (3)	0.3879 (2)	−0.42929 (16)	0.0742 (5)	
H17	0.5894	0.4283	−0.4275	0.089*	
N1	0.87874 (17)	0.27365 (13)	0.04012 (10)	0.0461 (3)	
O1	0.98538 (16)	0.15404 (13)	0.24616 (10)	0.0602 (3)	
O2	1.04747 (14)	0.03484 (12)	0.10209 (10)	0.0543 (3)	
O3	0.63449 (19)	0.57703 (15)	−0.30880 (11)	0.0789 (4)	
S1	0.92933 (5)	0.12195 (4)	0.15505 (3)	0.04628 (13)	

### Atomic displacement parameters (Å^2^)

**Table:**

	*U*^11^	*U*^22^	*U*^33^	*U*^12^	*U*^13^	*U*^23^
C1	0.0554 (8)	0.0428 (8)	0.0381 (7)	0.0021 (7)	−0.0076 (6)	−0.0064 (6)
C2	0.0723 (11)	0.0626 (11)	0.0470 (9)	−0.0010 (9)	0.0007 (8)	−0.0146 (8)
C3	0.0696 (12)	0.0795 (14)	0.0578 (11)	0.0037 (10)	0.0110 (9)	−0.0082 (9)
C4	0.0579 (10)	0.0687 (13)	0.0827 (14)	−0.0089 (9)	−0.0036 (10)	0.0032 (9)
C5	0.0669 (11)	0.0698 (13)	0.0848 (14)	−0.0156 (10)	−0.0104 (10)	−0.0201 (11)
C6	0.0619 (10)	0.0604 (11)	0.0593 (10)	−0.0069 (8)	−0.0060 (8)	−0.0209 (8)
C14	0.0447 (7)	0.0433 (8)	0.0444 (7)	−0.0093 (6)	−0.0023 (6)	−0.0140 (6)
C13	0.0606 (9)	0.0553 (10)	0.0513 (9)	−0.0105 (8)	−0.0034 (7)	−0.0234 (8)
C12	0.0666 (10)	0.0632 (12)	0.0739 (12)	−0.0113 (9)	−0.0011 (9)	−0.0391 (10)
C11	0.0712 (11)	0.0486 (10)	0.0844 (13)	−0.0044 (8)	−0.0049 (10)	−0.0280 (10)
C10	0.0611 (10)	0.0473 (9)	0.0632 (10)	−0.0049 (8)	−0.0074 (8)	−0.0107 (8)
C9	0.0440 (7)	0.0465 (8)	0.0456 (8)	−0.0096 (6)	−0.0016 (6)	−0.0115 (6)
C8	0.0483 (8)	0.0496 (9)	0.0386 (7)	−0.0083 (6)	−0.0041 (6)	−0.0105 (6)
C7	0.0535 (8)	0.0499 (9)	0.0359 (7)	−0.0072 (7)	−0.0052 (6)	−0.0149 (6)
C15	0.0546 (8)	0.0571 (10)	0.0403 (8)	−0.0080 (7)	−0.0082 (7)	−0.0053 (7)
C16	0.0677 (10)	0.0550 (10)	0.0337 (7)	−0.0120 (8)	−0.0094 (7)	−0.0041 (7)
C21	0.0728 (11)	0.0691 (12)	0.0399 (8)	−0.0044 (9)	−0.0087 (8)	−0.0138 (8)
C20	0.1019 (15)	0.0786 (14)	0.0471 (10)	0.0032 (12)	0.0000 (10)	−0.0194 (9)
C19	0.1479 (17)	0.0736 (14)	0.0441 (9)	−0.0172 (13)	−0.0036 (12)	−0.0223 (10)
C18	0.1306 (15)	0.0951 (17)	0.0512 (10)	−0.0334 (13)	−0.0220 (12)	−0.0222 (11)
C17	0.0873 (13)	0.0821 (14)	0.0465 (9)	−0.0182 (11)	−0.0203 (9)	−0.0097 (9)
N1	0.0566 (7)	0.0434 (7)	0.0357 (6)	−0.0037 (6)	−0.0071 (5)	−0.0132 (5)
O1	0.0728 (7)	0.0643 (7)	0.0457 (6)	−0.0020 (6)	−0.0197 (5)	−0.0220 (5)
O2	0.0551 (6)	0.0540 (7)	0.0498 (6)	0.0039 (5)	−0.0072 (5)	−0.0205 (5)
O3	0.0817 (9)	0.0791 (9)	0.0539 (7)	0.0174 (7)	−0.0184 (6)	−0.0119 (7)
S1	0.0526 (2)	0.0470 (2)	0.03579 (19)	0.00011 (16)	−0.00991 (15)	−0.01356 (15)

### Geometric parameters (Å, °)

**Table:**

C1—C2	1.381 (2)	C10—H10	0.9300
C1—C6	1.386 (2)	C9—C8	1.444 (2)
C1—S1	1.7498 (17)	C8—C7	1.356 (2)
C2—C3	1.384 (3)	C8—C15	1.468 (2)
C2—H2	0.9300	C7—N1	1.3913 (18)
C3—C4	1.375 (3)	C7—H7	0.9300
C3—H3	0.9300	C15—O3	1.227 (2)
C4—C5	1.364 (3)	C15—C16	1.490 (2)
C4—H4	0.9300	C16—C21	1.379 (2)
C5—C6	1.376 (3)	C16—C17	1.387 (2)
C5—H5	0.9300	C21—C20	1.385 (3)
C6—H6	0.9300	C21—H21	0.9300
C14—C13	1.387 (2)	C20—C19	1.385 (3)
C14—C9	1.397 (2)	C20—H20	0.9300
C14—N1	1.4152 (19)	C19—C18	1.369 (3)
C13—C12	1.370 (2)	C19—H19	0.9300
C13—H13	0.9300	C18—C17	1.361 (3)
C12—C11	1.388 (3)	C18—H18	0.9300
C12—H12	0.9300	C17—H17	0.9300
C11—C10	1.366 (3)	N1—S1	1.6677 (12)
C11—H11	0.9300	O1—S1	1.4195 (11)
C10—C9	1.399 (2)	O2—S1	1.4195 (11)
			
C2—C1—C6	121.26 (17)	C7—C8—C9	107.38 (13)
C2—C1—S1	119.33 (14)	C7—C8—C15	127.21 (15)
C6—C1—S1	119.39 (12)	C9—C8—C15	125.29 (15)
C1—C2—C3	118.46 (19)	C8—C7—N1	109.68 (13)
C1—C2—H2	120.8	C8—C7—H7	125.2
C3—C2—H2	120.8	N1—C7—H7	125.2
C4—C3—C2	120.39 (18)	O3—C15—C8	119.99 (16)
C4—C3—H3	119.8	O3—C15—C16	119.61 (14)
C2—C3—H3	119.8	C8—C15—C16	120.39 (14)
C5—C4—C3	120.5 (2)	C21—C16—C17	118.93 (18)
C5—C4—H4	119.7	C21—C16—C15	123.05 (15)
C3—C4—H4	119.7	C17—C16—C15	117.84 (17)
C4—C5—C6	120.5 (2)	C16—C21—C20	120.24 (18)
C4—C5—H5	119.8	C16—C21—H21	119.9
C6—C5—H5	119.8	C20—C21—H21	119.9
C5—C6—C1	118.91 (18)	C21—C20—C19	119.5 (2)
C5—C6—H6	120.5	C21—C20—H20	120.2
C1—C6—H6	120.5	C19—C20—H20	120.2
C13—C14—C9	122.50 (15)	C18—C19—C20	120.2 (2)
C13—C14—N1	130.70 (14)	C18—C19—H19	119.9
C9—C14—N1	106.78 (13)	C20—C19—H19	119.9
C12—C13—C14	117.03 (16)	C17—C18—C19	120.1 (2)
C12—C13—H13	121.5	C17—C18—H18	120.0
C14—C13—H13	121.5	C19—C18—H18	120.0
C13—C12—C11	121.58 (17)	C18—C17—C16	121.0 (2)
C13—C12—H12	119.2	C18—C17—H17	119.5
C11—C12—H12	119.2	C16—C17—H17	119.5
C10—C11—C12	121.37 (17)	C7—N1—C14	108.35 (12)
C10—C11—H11	119.3	C7—N1—S1	123.27 (10)
C12—C11—H11	119.3	C14—N1—S1	126.91 (10)
C11—C10—C9	118.68 (17)	O2—S1—O1	120.91 (7)
C11—C10—H10	120.7	O2—S1—N1	105.43 (7)
C9—C10—H10	120.7	O1—S1—N1	106.66 (7)
C14—C9—C10	118.82 (15)	O2—S1—C1	108.92 (7)
C14—C9—C8	107.78 (14)	O1—S1—C1	109.22 (7)
C10—C9—C8	133.37 (15)	N1—S1—C1	104.41 (7)
			
C6—C1—C2—C3	−0.4 (3)	C8—C15—C16—C21	−42.0 (2)
S1—C1—C2—C3	178.44 (13)	O3—C15—C16—C17	−35.9 (2)
C1—C2—C3—C4	0.2 (3)	C8—C15—C16—C17	142.89 (16)
C2—C3—C4—C5	0.2 (3)	C17—C16—C21—C20	1.0 (3)
C3—C4—C5—C6	−0.5 (3)	C15—C16—C21—C20	−174.08 (16)
C4—C5—C6—C1	0.3 (3)	C16—C21—C20—C19	−1.4 (3)
C2—C1—C6—C5	0.2 (3)	C21—C20—C19—C18	1.1 (3)
S1—C1—C6—C5	−178.69 (14)	C20—C19—C18—C17	−0.4 (4)
C9—C14—C13—C12	0.7 (2)	C19—C18—C17—C16	0.0 (3)
N1—C14—C13—C12	178.95 (15)	C21—C16—C17—C18	−0.3 (3)
C14—C13—C12—C11	−1.5 (3)	C15—C16—C17—C18	175.06 (18)
C13—C12—C11—C10	1.0 (3)	C8—C7—N1—C14	1.51 (16)
C12—C11—C10—C9	0.3 (3)	C8—C7—N1—S1	168.55 (10)
C13—C14—C9—C10	0.5 (2)	C13—C14—N1—C7	−179.63 (15)
N1—C14—C9—C10	−178.11 (13)	C9—C14—N1—C7	−1.22 (16)
C13—C14—C9—C8	179.08 (14)	C13—C14—N1—S1	13.9 (2)
N1—C14—C9—C8	0.51 (16)	C9—C14—N1—S1	−167.65 (10)
C11—C10—C9—C14	−1.0 (2)	C7—N1—S1—O2	33.46 (13)
C11—C10—C9—C8	−179.16 (16)	C14—N1—S1—O2	−161.98 (12)
C14—C9—C8—C7	0.40 (16)	C7—N1—S1—O1	163.18 (12)
C10—C9—C8—C7	178.74 (17)	C14—N1—S1—O1	−32.27 (14)
C14—C9—C8—C15	176.58 (14)	C7—N1—S1—C1	−81.25 (13)
C10—C9—C8—C15	−5.1 (3)	C14—N1—S1—C1	83.30 (13)
C9—C8—C7—N1	−1.18 (17)	C2—C1—S1—O2	155.20 (12)
C15—C8—C7—N1	−177.26 (14)	C6—C1—S1—O2	−25.92 (15)
C7—C8—C15—O3	161.36 (17)	C2—C1—S1—O1	21.21 (15)
C9—C8—C15—O3	−14.1 (2)	C6—C1—S1—O1	−159.91 (13)
C7—C8—C15—C16	−17.5 (2)	C2—C1—S1—N1	−92.56 (13)
C9—C8—C15—C16	167.13 (14)	C6—C1—S1—N1	86.32 (14)
O3—C15—C16—C21	139.19 (18)		

### Hydrogen-bond geometry (Å, °)

**Table:**

Cg1 is the centroid of the C16–C21 ring.

**Table:**

*D*—H···*A*	*D*—H	H···*A*	*D*···*A*	*D*—H···*A*
C2—H2···O1	0.93	2.59	2.934 (2)	103
C10—H10···O3	0.93	2.57	3.068 (3)	114
C13—H13···O1	0.93	2.42	2.999 (2)	120
C6—H6···O2^i^	0.93	2.58	3.493 (2)	167
C7—H7···O2^i^	0.93	2.54	3.429 (2)	160
C4—H4···Cg1^ii^	0.93	2.98	3.774 (3)	144

Symmetry codes: (i) −*x*+2, −*y*, −*z*; (ii) −*x*+1, −*y*, −*z*.
